# Imaging Modalities to Assess Oxygen Status in Glioblastoma

**DOI:** 10.3389/fmed.2015.00057

**Published:** 2015-08-19

**Authors:** Aurélien Corroyer-Dulmont, Ararat Chakhoyan, Solène Collet, Lucile Durand, Eric T. MacKenzie, Edwige Petit, Myriam Bernaudin, Omar Touzani, Samuel Valable

**Affiliations:** ^1^CNRS, UMR 6301-Imagerie et stratégies thérapeutiques des pathologies cérébrales et tumorales (ISTCT), CERVOxy group, GIP Cyceron, Caen, France; ^2^CEA, Direction des Sciences du Vivant (DSV)/Institut d’Imagerie Biomédicale (I2BM), UMR 6301-Imagerie et stratégies thérapeutiques des pathologies cérébrales et tumorales (ISTCT), CERVOxy group, GIP Cyceron, Caen, France; ^3^Université de Caen Normandie, UMR 6301-Imagerie et stratégies thérapeutiques des pathologies cérébrales et tumorales (ISTCT), CERVOxy group, GIP Cyceron, Caen, France; ^4^Esplanade de la Paix, Normandie Université, Caen, France

**Keywords:** hypoxia, glioblastoma, MRI, PET, multimodal imaging

## Abstract

Hypoxia, the result of an inadequacy between a disorganized and functionally impaired vasculature and the metabolic demand of tumor cells, is a feature of glioblastoma. Hypoxia promotes the aggressiveness of these tumors and, equally, negatively correlates with a decrease in outcome. Tools to characterize oxygen status are essential for the therapeutic management of patients with glioblastoma (i) to refine prognosis, (ii) to adapt the treatment regimen, and (iii) to assess the therapeutic efficacy. While methods that are focal and invasive in nature are of limited use, non-invasive imaging technologies have been developed. Each of these technologies is characterized by its singular advantages and limitations in terms of oxygenation status in glioblastoma. The aim of this short review is, first, to focus on the interest to characterize hypoxia for a better therapeutic management of patients and, second, to discuss recent and pertinent approaches for the assessment of oxygenation/hypoxia and their direct implication for patient care.

## Hypoxia and Glioblastoma

**Figure 1:**
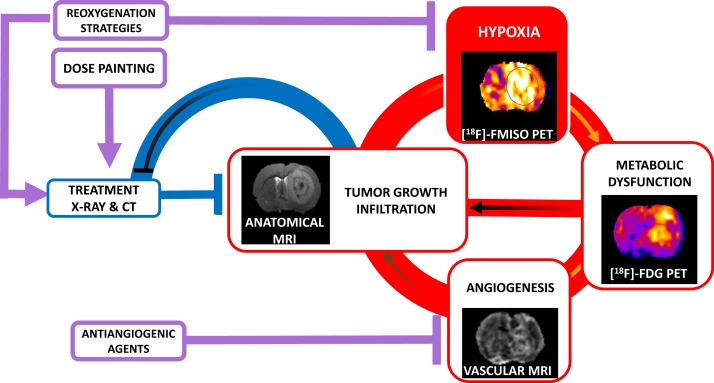
**Schematic representation of the negative role of hypoxia in tumor growth and therapeutic response in glioblastoma**. The growth of a GBM is a feed-forward (vicious) cycle exacerbated by hypoxia as depicted in red. Hypoxia renders the tumor less susceptible to standard chemo-(CT) and radio-therapy (X-ray) as shown in blue. Future treatment strategies are indicated in purple.

As an aggressive solid tumor, glioblastoma (GBM) presents cardinal histological features of chaotic microvascular proliferation and necrotic foci. However, the neoangiogenesis is dysfunctional and normal tissue oxygen tension is not maintained (1). The resulting imbalance between oxygen consumption and supply is called hypoxia. An important spatial heterogeneity is observed in GBM with severely hypoxic zones and peripheral infiltration accompanied by a microvascular proliferation (2). GBM cells, in hypoxic conditions, express transcriptional factors, such as the hypoxia-inducible factor (HIF), which, by the induction of various gene expressions, is involved in tumor cell adaptation. Hypoxia plays a pivotal role in the spread of tumor cells and, hence, invasion (3). Hypoxia is also a key factor in the rapaciousness of human GBM and the characterization of hypoxia discriminates poor from long outcomes (4). Moreover, hypoxia greatly impacts therapeutic efficacy. For instance, it is known that the degree of oxygenation is critical for the efficacy of both radiation therapy and chemotherapy (5). All these aspects are summarized in Figure 1. Hence, the assessment of tissue oxygen tension in GBM is crucial to personalize the treatment for each patient and to monitor its efficacy. Various methods, more or less invasive (Table 1), have been developed to assess the level of oxygenation at different compartments ranging from blood to cells.

**Table 1:** **Methodological approaches to evaluate oxygenation in glioblastoma: advantages and limitations**.

	Advantages	Limitations
Immunohistochemistry	Routinely performed in neuropathology labs	Indirect assessment of p_t_O_2_
		Invasive and localized
Probes	Direct and quantitative assessment of p_t_O_2_	Invasive and localized
		Low reproducibility
		Consumption of oxygen (for Eppendorf probes)
NIRS	Setup and application in clinical routine are easy	Indirect assessment of p_t_O_2_
	No contrast agent injection	Superficial and regional
qBOLD	Setup and application in clinical routine are easy	Indirect assessment of p_t_O_2_
	Whole brain characterization	Specificity for hypoxia needs to be validated
	Sensitive	
[^19^F]-MRI	Direct and quantitative assessment of p_t_O_2_	Needs contrast agent injection potentially toxic
		Needs ^19^F coil (not easy for clinic application)
		Relatively low spatial resolution
EPR imaging	Direct and quantitative assessment of p_t_O_2_	Localized (for EPR)
	Whole brain characterization	Very low spatial resolution
		No studies in brain tumors
MOBILE	Whole brain characterization	Indirect and relative assessment of p_t_O_2_
		No studies in brain tumors
Bioluminescence imaging	Indicator of cellular hypoxia	Needs genetically engineered tumor cells
		Not applicable for patients
^15^Oxygen	Whole brain characterization	Administration of a gaseous radioactive contrast agent
		No linear relation between oxygen consumption and cellular hypoxia
[^62^Cu]/[^64^Cu]-ATSM	Characterization of moderate hypoxia	Injection of a radioactive contrast agent
	Whole brain characterization	Long half-life (12.7 h)
		Specificity for hypoxia is discussed
[^18^F]-FMISO	Indicator of cellular hypoxia	Injection of a radioactive contrast agent
	Particularly adapted to radiation therapy modulation	Relative long time before steady-state acquisition (2 h)
	Whole brain characterization	
[^18^F]-FAZA	Indicator of cellular hypoxia	Injection of a radioactive contrast agent
	More rapid clearance than [^18^F]-FMISO	Needs to be validated in a greater number of studies
	Whole brain characterization	
[^18^F]-HX4 and [^18^F]-FETNIM	Indicator of cellular hypoxia	Injection of a radioactive contrast agent
	Whole brain characterization	Not recommended for brain tumors
[^18^F]-EF5	Indicator of cellular hypoxia	Injection of a radioactive contrast agent
	Sensitivity near to immunohistochemistry	Synthesis of radiotracer remains difficult
	Whole brain characterization	
[^18^F]-RP-170	Reflect of cellular hypoxia	Injection of a radioactive contrast agent
	Whole brain characterization	Needs to be validated in a greater number of studies

## Identification of Hypoxia at the Molecular Scale: Immunohistochemistry

Evaluation of hypoxia at the molecular scale can be performed by the detection of hypoxia-activated endogenous molecules, such as HIFα, and its targets, such as CAIX, Glut1, and VEGF. Another approach is to detect pimonidazole uptake in cells after systemic administration prior to biopsy. Immunohistochemistry has demonstrated its usefulness in the classification of GBM patients as a function of the level of hypoxia within the tumor and then predict recurrence and overall survival (4). The limitation to this technique is its invasive nature and the approach is restricted to a small biopsy, which may be not representative of the entire tumoral mass. Ideally and given the temporo-spatial heterogeneity of GBM, a method able to continuously and repeatedly assess hypoxia by three-dimensional imaging would be more than desirable.

## Assessment of Local Tissue Oxygenation: Intracerebral Probes

The most direct assessment of tissue oxygenation is to measure the regional tissular oxygen pressure (p_t_O_2_) by probes implanted directly into the brain. The first probe developed was the Eppendorf polarographic p_t_O_2_ electrode, which relies on the measurement of the current generated at a cathode coated with thin gold by ionization of oxygen at −700 mV. The needle is mounted on a stepping motor that sequentially advances and retracts the needle tip. This approach allows the creation of a histogram of p_t_O_2_ recorded from the tissue of interest. The temporal assessment of p_t_O_2_ is possible but the electrochemical reduction reduces the dissolved oxygen causing a continuous signal decrease with time. A second probe, more recently used, is the OxyLite™ probe (Oxford-Optronix). Light pulses carried by an optical fiber induce pulsatile fluorescence of a ruthenium luminophore incorporated into a silicone rubber polymer at the probe tip. The lifetime of the fluorescent pulses is inversely proportional to the oxygen tension in the tip. In a syngeneic model of rhabdomyosarcoma, the sensitivity of this method was exemplified by a real-time variation of tumor p_t_O_2_ during induced hypercapnia (6).

Despite the fact that oxygen sensitive probes allow a direct assessment of p_t_O_2_, their major limitation is that they only allow a focal measurement of O_2_ tensions. It is not possible to ascertain the exact location of the probe, e.g., in a necrotic or a peripheral area of the tumor. A second disadvantage is the invasiveness of these probes, which are unlikely to have clinical applications. Finally, there is also the fact that histography consumes oxygen.

## Assessment of Hypoxia in Glioblastoma with Imaging

Several imaging methods have been developed to assess oxygen status in GBM patients but two main approaches are widely employed and based on MRI and PET technologies. These methods evaluate the level of oxygen in different physiological compartments: blood vessels (oxygen saturation, SO_2_), tissue (p_t_O_2_), or cellular level and can thus render directly or indirectly indices of oxygenation.

## Estimation of Tumor Hypoxia in the Vascular Compartment: NIRS and BOLD-MRI

### Near infrared spectroscopy

Near infrared spectroscopy (NIRS) is a simple, non-invasive but indirect method to characterize the level of oxygenation in blood. Hemoglobin (Hb) has an important photon absorption level in the near infrared range. The absorption spectrum is different if the hemoglobin is fully oxygenated or fully deoxygenated (deoxyHb). NIRS is able to characterize the concentration of the different forms of hemoglobin to reflect oxygen saturation (7). However, the NIRS characterization is superficial and is only reliable to a depth of 3–5 mm in the adult.

### Blood oxygen level-dependent MRI

Blood oxygen level-dependent MRI (BOLD-MRI) is a tomographic approach to detect changes in paramagnetic deoxyHb. From the complex relationship between T2* and the Hb/deoxyHb ratio, a method to measure the SO_2_ has been developed and termed as qBOLD-MRI where “q” stands for quantitative. The relationship between the T2* signal and SO_2_ is multifactorial and depends on several parameters, such as cerebral blood volume (CBV), local hematocrit, T2 value, *B*_0_ field, and oxygen saturation (8). With the quantification and integration of these parameters, a SO_2_ map can be obtained. At a preclinical level, Christen and colleagues (8) have demonstrated the sensitivity of this method to detect changes not only in brain oxygenation during hypoxemia episodes but also in murine orthotopic models of GBM (9). Given that anatomical MRI is in use routinely, additional scan time to characterize tumor hypoxia by qBOLD-MRI could yield meaningful clinical data. The ready accessibility of MRI tempts one to use BOLD-MRI to assess hypoxia. However, BOLD-MRI, entirely focused on the vascular compartment (SO_2_), corresponds to an indirect assessment of oxygenation in tissue. While the use of qBOLD-MRI corrects some pitfalls in the standard BOLD-MRI technique (10), the specificity of this approach for hypoxia still requires validation. Indeed, SO_2_ and p_t_O_2_ are non-linear and various parameters affect the sigmoid shape of the relationship between the two parameters. Nonetheless, the extrapolation of p_t_O_2_ from SO_2_ is feasible under physiological circumstances, it is highly critical in a tumoral environment with several disturbed metabolic processes.

## Imaging of Oxygenation in the Tissue Compartment: ^19^F-MRI and EPRI

### [^19^F]-MRI

The relaxivity (R1) of contrast agents saturated with ^19^fluorine (such as perfluorocarbons or hexafluorobenzene administered either intravenously or directly in the brain tissue) is directly proportional to p_t_O_2_. In a murine GBM model, Lemaire and colleagues (11) have shown the ability of [^19^F]-MRI to characterize the difference of tumor oxygenation between basal conditions (p_t_O_2_ ≈ 10 mmHg) and during inhalation of 100% oxygen (p_t_O_2_ ≈ 170 mmHg). Although [^19^F]-MRI is quantitative, this approach is limited by the potential toxicity of the tracer.

### Electron paramagnetic resonance imaging

Electron paramagnetic resonance (EPR) specifically responds only to atoms or molecules with unpaired electrons, including free radicals, free electrons, and some valence states of metal ions. The phenomenon that is observed with EPR is the transition (resonance absorption of energy) between the two energy states that can occur in an unpaired electron system placed within a magnetic field. This field separates the energy states associated with the two possible spin states of unpaired electrons. The presence of other unpaired electron species can affect the EPR spectrum. Molecular oxygen, with two unpaired electrons, can be detected either directly by EPR at ambient conditions due to fast relaxation of spins or detected indirectly from the oxygen-induced changes in the EPR spectrum of other paramagnetic probes in the system. From oxygen-induced EPR line bandwidth, one can quantify the oxygen concentration (12), in a preclinical study, have shown the ability and repeatability of electron paramagnetic resonance imaging (EPRI) to evaluate the different levels of oxygen in four brain tumor models. Furthermore, EPRI permits a spatial localization of p_t_O_2_ (13). However, because the signal is weak in EPRI, the slice thickness is important and the spatial resolution relatively low.

## Assessment of Oxygenation at the Cellular Level: MOBILE and BLI

### Mapping of oxygen by imaging lipid relaxation enhancement-MRI

Because oxygen is 11 times more soluble in lipids than in water, it has been proposed to measure T1 relaxivity in lipids for the assessment of oxygen status (14). With GBM, this method holds promise given that the cerebral concentration of phospholipids is important in the brain. However, even if this method is sensitive to changes in p_t_O_2_ during gas inhalation, for example, studies in brain tumors have yet to be performed.

### Bioluminescence imaging

Bioluminescence imaging (BLI) is based on the detection of light emitted by cells expressing a luciferase gene under the control of a hypoxia-sensitive promoter containing hypoxia response elements (HRE) sequences, has been advanced to measure brain hypoxia. An engineered human GBM cell line expressing luciferase under the control of HRE was implanted orthotopically and well correlated with other hypoxic reporters (15). Nonetheless, BLI has a low-spatial resolution and the tumoral heterogeneity will be difficult to assess and no translation to man is feasible.

## Assessment of Hypoxia with PET

### ^15^Oxygen

The first PET radiotracer, used to assess oxygen levels in brain, was the positron emitter ^15^Oxygen which allowed several validated quantitative methods to map cerebral blood flow (CBF), CBV, oxygen extraction fraction (OEF), and the cerebral metabolic rate of oxygen (CMRO_2_) using ^15^O-labeled H_2_O, CO, and O_2_ (16). These methods are considered as the “gold standard” and are widely employed, among others, in the study of stroke.

However, these methods are not immediately translatable to oncology. Tumor metabolism is driven by the rate of glycolysis but without a corresponding increase in aerobic metabolism (the Warburg effect). In neoplasm, the inability of these techniques to reflect neither p_t_O_2_ nor true CMRO_2_ has led to the development of various other PET tracers better adapted to this end. Two classes of radiotracers are employed: the 62/64Cu labeled-diacetyl-bis(N4-methylthiosemicarbazone) and ^18^F-labeled nitroimidazoles analogs, discussed as follows.

### Cu-diacetyl-bis(N4-methylthiosemicarbazone) ([^64^Cu] or [^62^Cu]-ATSM)

[^62^/^64^Cu]-ATSM has been promoted, not as an oxygen sensor, but as a hypoxia biomarker. Cu(II)-ATSM is reduced to an unstable Cu(I)-ATSM and then re-oxidized if oxygen is present but otherwise becomes trapped in hypoxic cells. This radiotracer has the capability to characterize moderate hypoxia with an enhanced uptake observed when p_t_O_2_ is <35 mmHg (17). The long half-life of [^64^CU]-ATSM (12.7 h) is an advantage because production and transport would not only permit utilization in different clinical research centers but also a limitation in terms of radioprotection and the impossibility to repeat the measure more than once a week. With respect to neurooncology, Tateishi and colleagues (18) examined the interest of using [^62^Cu]-ATSM for glioma grading and correlated [^62^Cu]-ATSM uptake with HIF-1α expression in glioma patients. However, several studies have suspected a non-specificity of this radiotracer in hypoxia with an absence of correlation between the spatial distribution of [^64^Cu]-ATSM uptake immunohistochemical characterization of hypoxia (19).

### Fluorinated nitroimidazole compounds

Nitroimidazoles enter viable cells by passive diffusion where they undergo an active reduction. Under normoxic conditions, these molecules are re-oxidized and diffuse out of the cell. By contrast, in severe hypoxia, they eventually become irreversibly trapped in the cell at a threshold p_t_O**_2_** <10 mmHg. Several nitroimidazole radiotracers are at various stages of preclinical and clinical development.

### [^18^F]-fluoromisonidazole ([^18^F]-FMISO)

Highly selective for hypoxia, [^18^F]-FMISO is the lead candidate to assess hypoxia with PET and is the most extensively studied radiotracer in clinical investigations. In high-grade glioma, where the p_t_O_2_ in the tumor is remarkably low (around 7 mmHg), [^18^F]-FMISO is increasingly used to estimate hypoxia (20). In our group, we have shown the ability of [^18^F]-FMISO to discriminate various states of hypoxia in different GBM models (21, 22). Others studies have shown that the uptake of [^18^F]-FMISO is inversely correlated to overall survival in GBM patients (23). The major limitation of this approach is the low sensitivity to moderate hypoxia (between 10 and 30 mmHg). The second limitation of this radiotracer is its relative long washout in non-hypoxic cells to obtain an optimal contrast (between 2 and 4 h after tracer injection).

### Other hydrophilic tracers [^18^F]-fluoroazomycin-arabinoside ([^18^F]-FAZA), [^18^F]-flortanidazole ([^18^F]-HX4) and [^18^F]-fluoroerythronitroimidazole ([^18^F]-FETNIM), 1-[2-[^18^F] fluoro-1-(hydroxymethyl)-ethoxy]methyl-2-nitroimidazole ([^18^F]-RP-170)

These tracers ([^18^F]-Fluoroazomycin-arabinoside ([^18^F]-FAZA), [^18^F]-Flortanidazole ([^18^F]-HX4), and [^18^F]-fluoroerythronitro imidazole ([^18^F]-FETNIM), 1-[2-[^18^F] Fluoro-1-(hydroxyme thyl)-ethoxy]methyl-2-nitroimidazole ([^18^F]-RP-170) because of their hydrophilic character, have a more rapid plasma half-life and rapid clearance from tissues than [^18^F]-FMISO. [^18^F]-FAZA has been used to determine hypoxia in models of gliomas and discriminated clusters of hypoxia and necrosis (24). [^18^F]-FAZA awaits clinical investigation for GBM patients. In glioma patients, [^18^F]-RP-170 was compared to p_t_O_2_ and HIF-1α immunostaining and a selectivity of this tracer for hypoxia was observed (25). However, regarding their highly hydrophilic properties, these radiotracers are not recommended the study of brain tumors because their uptake will be largely influenced by BBB disruption (26).

### 2-(2-nitro-1H-imidazol-1-yl)-N-(2,2,3,3,3-penta-fluoropropyl)-acetamide ([^18^F]-EF5)

In contradistinction to the hydrophilic tracers, a lipophilic radiotracer, [^18^F]-EF5, has been studied to detect hypoxia in human GBM. Initially, it was used for immunohistochemistry, [^18^F]-EF5 possesses a sensitivity near to that of immunohistochemistry but the synthesis seems problematic (27).

## Interest of PET Tracers in the Management of GBM Patients

The conventional treatment of GBM is surgical resection when possible, followed by radiotherapy along with concomitant chemotherapy. Despite these treatment protocols, the median survival fails to exceed 15 months. Imaging of hypoxia should ameliorate patient management in various ways (see Figure 1).

### Optimization of conventional treatments

In the therapeutic management of glioma patients, it has been proposed to use imaging biomarkers of hypoxia to adapt the treatment according to hypoxic status (20). [^18^F]-FMISO seems particularly suited for the modulation of radiation dose in GBM because the range of p_t_O_2_ identified by [^18^F]-FMISO (from 0 to 10 mmHg) corresponds to the scale used to define the radiation dose adapted for hypoxic, radioresistant tumors (28). Hence, authors have advanced the use of the spatial information derived from [^18^F]-FMISO to compute the prescription for the radiation dose (29). The aim of dose escalation is (i) to increase the efficacy of treatment in hypoxic area, (ii) to compensate for the spatial heterogeneity, and (iii) to reduce the toxicity to healthy tissue. While a boost of radiotherapy in the most hypoxic areas appears attractive, this concept still requires validation.

### Treatment orientation

Glioblastoma is densely vascularized tumors and, therefore, targeted anti-angiogenic therapies, such as bevacizumab, (a humanized monoclonal antibody raised against VEGF), has been suggested, although recent phase III trials demonstrated disappointing results. An explanation for the failure could be that no patient selection was performed prior to the initiation of the treatment. PET-based imaging of hypoxia could be performed before treatment to stratify eligible patients for the anti-angiogenic strategy. After selection by markers of hypoxia, the use of radiosensitizers has also been proposed for head and neck cancers (EORCT trail 1219), an approach that could be applied to GBM patients. Our group has also demonstrated that hypoxia-inducible genes, such as erythropoietin, are involved in GBM growth and resistance to treatment (5). Grounded on hypoxia imaging, such proteins would represent novel and original targets for therapy.

## Assessment of Treatment Effects

The interest in anti-VEGF therapies has been rekindled since the emergence of the concept of “normalization.” Anti-angiogenic treatments are now planned to transiently improve tumor vasculature, resulting in increased tumor perfusion and oxygenation. In a preclinical study in GBM, we have shown that sunitinib treatment decreases [^18^F]-FMISO uptake hypothetically due to vascular normalization (21). Similarly, Titz and colleagues (30) have proposed to program a model of responsivity to anti-angiogenic treatments from PET imaging of hypoxia in patients.

## Conclusion

It is widely accepted that hypoxia is important not only in tumor growth but also in response to various therapeutic regimens. The characterization of oxygenation by non-invasive imaging remains a challenge for the management and adaptation of conventional treatments (radio- and chemotherapy) as well as to follow the efficacy of treatment. Several attempts have been made to develop methods to measure and image tumor oxygen tensions *in vivo* without consuming oxygen and thus without exacerbating the degree of hypoxia. Various approaches have been proposed to evaluate brain oxygenation at different scales from vascular to molecular levels. In this review, we argue that, despite development of several other methods (summarized Table 1), PET imaging (especially with fluorinated nitroimidazole compounds) seems to be, until now, the most relevant tool to characterize hypoxia in GBM.

## Author Contributions

Manuscript drafting or manuscript revision for important intellectual content: all authors. Manuscript final version approval: all authors. Literature research: AC-D, AC, SC, EM, OT, and SV. Manuscript editing: AC-D, EM, and SV. Figure preparation: AC-D, AC, EM, and SV.

## Conflict of Interest Statement

The authors declare that the research was conducted in the absence of any commercial or financial relationships that could be construed as a potential conflict of interest.

## Abbreviations

BLI, bioluminescence imaging; EPR, electron paramagnetic resonance; GBM, glioblastoma; HB, hemoglobin; HIF, hypoxia-inducible factor; MRI, magnetic resonance imaging; NIRS, near infrared spectroscopy; PET, positron emission tomography; p_t_O_2_, tissular oxygen pressure; SO_2_, oxygen saturation.
